# Analyzing the expression pattern of the noncoding RNAs (HOTAIR, PVT‐1, XIST, H19, and miRNA‐34a) in PBMC samples of patients with COVID‐19, according to the disease severity in Iran during 2022–2023: A cross‐sectional study

**DOI:** 10.1002/hsr2.1861

**Published:** 2024-02-07

**Authors:** Khadijeh Khanaliha, Javid Sadri Nahand, AliReza Khatami, Hamed Mirzaei, Sara Chavoshpour, Mohammad Taghizadieh, Mohammad Karimzadeh, Tahereh Donyavi, Farah Bokharaei‐Salim

**Affiliations:** ^1^ Research Center of Pediatric Infectious Diseases, Institute of Immunology and Infectious Diseases Iran University of Medical Sciences Tehran Iran; ^2^ Infectious and Tropical Diseases Research Center Tabriz University of Medical Sciences Tabriz Iran; ^3^ Department of Virology Iran University of Medical Sciences Tehran Iran; ^4^ Research Center for Biochemistry and Nutrition in Metabolic Diseases Kashan University of Medical Sciences Kashan Iran; ^5^ Department of Virology Tehran University of Medical Sciences Tehran Iran; ^6^ Department of Pathology, Faculty of Medicine Tabriz University of Medical Sciences Tabriz Iran; ^7^ Core Research Facilities (CRF) Isfahan University of Medical Science Isfahan Iran; ^8^ Department of Medical Biotechnology, Faculty of Allied Medicine Iran University of Medical Sciences Tehran Iran

**Keywords:** biomarker, long noncoding RNAs, miR‐34a, SARS‐CoV‐2, severe COVID‐19

## Abstract

**Background and aims:**

MicroRNAs (miRNAs) and long noncoding RNAs (lncRNAs) are well‐known types of noncoding RNAs (ncRNAs), which have been known as the key regulators of gene expression. They can play critical roles in viral infection by regulating the host immune response and interacting with genes in the viral genome. In this regard, ncRNAs can be employed as biomarkers for viral diseases. The current study aimed to evaluate peripheral blood mononuclear cell (PBMC) ncRNAs (lncRNAs‐homeobox C antisense intergenic RNA [HOTAIR], ‐H19, X‐inactive‐specific transcript [XIST], plasmacytoma variant translocation 1 [PVT‐1], and miR‐34a) as diagnostic biomarkers to differentiate severe COVID‐19 cases from mild ones.

**Methods:**

Candidate ncRNAs were selected according to previous studies and assessed by real‐time polymerase chain reaction in the PBMC samples of patients with severe coronavirus disease 2019 (COVID‐19) (*n* = 40), healthy subjects (*n* = 40), and mild COVID‐19 cases (*n* = 40). Furthermore, the diagnostic value of the selected ncRNAs was assessed by analyzing the receiver‐operating characteristic (ROC).

**Results:**

The results demonstrated that the expression pattern of the selected ncRNAs was significantly different between the studied groups. The levels of HOTAIR, XIST, and miR‐34a were remarkably overexpressed in the severe COVID‐19 group in comparison with the mild COVID‐19 group, and in return, the PVT‐1 levels were lower than in the mild COVID‐19 group. Interestingly, the XIST expression level in men with severe COVID‐19 was higher compared to women with mild COVID‐19. ROC results suggested that HOTAIR and PVT‐1 could serve as useful biomarkers for screening mild COVID‐19 from severe COVID‐19.

**Conclusions:**

Overall, different expression patterns of the selected ncRNAs and ROC curve results revealed that these factors can contribute to COVID‐19 pathogenicity and can be considered diagnostic markers of COVID‐19 severe outcomes.

## 1 INTRODUCTION

The emergence of the coronavirus disease 2019 (COVID‐19) pandemic caused by the severe acute respiratory syndrome coronavirus 2 (SARS‐CoV‐2) virus has so far caused unexpected deaths and imposed many health and economic losses around the world.^1^ Many studies have been and are still being performed to decipher the pathogenesis enigmas of the virus.^2^ The key problematic factor is the host immune system‐virus interaction, resulting in inflammation.^3^, ^4^, ^5^, ^6^ As far as it is known, inflammation severity depends on the virus strain, immune system status of the hosts, and viral load, as well as which tissues and organs are involved, such as the lung, heart, kidneys,^7^ and liver.^8^, ^9^ Numerous clinical symptoms, from mild to severe, are manifested.^10^, ^11^ From the intracellular mechanism and molecular levels point of views, the inflammation process is multifactorial and complex, which is controlled by the mediators.^12^

Despite the frequent cases of COVID‐19, there is still a lack of confirmed biomarkers that can accurately diagnose and predict the severity of the disease. Various studies have suggested potential biomarkers, including the prognostic nutritional index, the ratio of C‐reactive protein (CRP) to prealbumin, and high‐sensitivity CRP to albumin ratio, but they have not yet been fully validated.^13^ Elevated levels of CRP, fibrinogen‐to‐albumin ratio, D‐dimer, and tumor necrosis factor‐α, as well as decreased CD4+/CD8+ ratio, platelet count, lymphocyte count, leukocyte count, and lymphocyte count, have been proposed as potential biomarkers for measuring the severity of SARS‐CoV‐2 infection.^14^, ^15^

Noncoding RNAs (ncRNAs) constitute a majority of the human genome, and despite their lack of translation into a protein, they play crucial roles in diverse vital processes such as inflammation, differentiation, cell apoptosis, organogenesis, and the posttranscriptional levels.^16^, ^17^ The precise numbers of ncRNAs in the human genomes have not yet been determined; they are separated and known depending on their function and, mostly, their length.^18^, ^19^ Both short and long ncRNAs have been repeatedly associated with some diseases and the control of gene expression, particularly in immune cells.^20^ Nowadays, long noncoding RNAs (lncRNAs) have been the research hotspot of the disease diagnosis and/or prognosis field; diagnosis via noninvasive biomarkers that can be recovered from body fluids, such as lncRNA, is a hot topic that also applies to COVID‐19, so that with the changes in the expression levels, assessments of diagnostic targets can be used to monitor and prognoses the disease more quickly and adopt the appropriate treatment strategies.^21^, ^22^, ^23^, ^24^

Dispersive literature has revealed the potential role of RNAs in immune responses and inflammatory signaling against COVID‐19 infection; they are indirectly responsible for the infection's clinical manifestations.^25^ *HOTAIR*, as a well‐known lncRNA, participates in various molecular mechanisms and has been considered necessary for the expression of pro‐inflammatory factors induced by lipopolysaccharide.^26^, ^27^ Plasmacytoma variant translocation 1 (PVT‐1) has an inflammatory response and regulation ability through multiple pathways.^28^ The possibility of the participation of lncRNA in pulmonary diseases is also raised, and this feature can be significant in COVID‐19.^29^ Similarly, the lncRNA X‐inactive‐specific transcript (XIST), as a member of the circulatory lncRNA family, has been frequently associated with several diseases and indicates the high clinical diagnostic potential for pro‐inflammatory and inflammatory responses.^30^, ^31^ Finally, as one of the oldest lncRNAs discovered, the lncRNA H19 participates in the regulation of inflammatory cytokine levels, pulmonary fibrosis, and lung inflammation, which are COVID‐19 complications.^32^, ^33^ MicroRNA (miR)‐34a, as a highly conserved small‐size ncRNA class, has been associated with apoptosis, (anti) cancers, and inflammation processes, and studies have also revealed the relationship with lncRNA‐XIST.^34^, ^35^ Our research has mainly sought to assess the mentioned ncRNA expression levels in people with COVID‐19 according to the severity of their clinical symptoms.

## 2 MATERIALS AND METHODS

### 2.1 Population and ethical issues

From January 2022 to February 2023, 80 COVID‐19 patients referred to Hazrat Rasoul Akram Hospital in Tehran (Iran) were included in this cross‐sectional survey. Forty of the patients did not present complications and were treated outpatient, 40 patients were hospitalized due to severe complications, and 40 healthy people were enrolled as a control group.

People who had simultaneous infections with hepatitis B and/or C viruses, human immunodeficiency virus, influenza, or *Mycobacterium tuberculosis* were excluded from the study. No participants had underlying medical conditions, with the exception of diabetes.

The Ethics Committee of Iran University of Medical Sciences (IUMS), with the ethical code: IR. IUMS. REC.1401.571, verified the present survey. All the volunteers for the current research filled out the consent form.

### 2.2 Peripheral blood mononuclear cell (PBMC) preparation

A total of 5 mL of peripheral blood specimens were collected from all the respondents into a Vacutainer tube with ethylenediamine tetraacetic acid. Then, PBMCs were isolated using the Ficoll‐Paque density gradient centrifugation method.^36^ Finally, the PBMC pellet was dissolved in 300 μL of RNAlater solution (SIGMA R 0901) and frozen at −70°C until the isolation of the total RNA.

### 2.3 Expression analysis of miR‐34a

The total RNA of PBMCs was extracted using the miRNeasy Mini Kit (Qiagen) and following the manufacturer's protocol. The quality and purity of the isolated RNA were measured with a NanoDrop spectrophotometer (Thermo Scientific Inc.) and subsequently stored at −20°C. In addition, the miScript® II RT (Qiagen) Kit was used to synthesize complementary DNA (cDNA) on 5 μg of the total RNA, and then the expression pattern of miR‐34a^37^ was evaluated in two groups of cases with COVID‐19 (according to the disease severity) and healthy people those absences of respiratory tract infection or COVID‐19 specifically.

Relative quantification by real‐time polymerase chain reaction (RT‐PCR) was performed using the miScript SYBR Green PCR Kit, based on the manufacturer's procedures, with a Rotor‐Gene Q instrument (QIAGEN GmbH) system. All RT‐PCR reactions were conducted in triplicate for each sample. This experiment was performed according to certain conditions, namely, 2 min at 95°C, followed by 40 cycles at 95°C for 20 s, at 60°C for 25 s, and at 70°C for 30 s. The housekeeping gene SNORD47 was set as a reference gene to normalize miR‐34a expression levels. Normalized fold change was measured by the $2^{‐∆∆C_{t}}$ formula compared to the healthy control group.

### 2.4 Expression pattern analysis of lncRNAs

To determine the expression level of the selected lncRNAs (homeobox C antisense intergenic RNA [HOTAIR],^38^ plasmacytoma variant translocation 1 [PVT‐1],^39^ X‐inactive‐specific transcript [XIST],^37^ and H19,^40^ Table 1), cDNA was synthesized using 350 ng of the extracted RNA, which has been presented completely.^41^

**Table 1:** Primers used in the present study for determining of expression profile of noncoding RNAs.

Sequences	Name	Real‐time PCR based on SYBR‐Green I fluorescence
5′‐GGGGTGGCAGTGTCTTAGC‐3′	MiR‐34a‐F	Real‐time PCR for MiR‐34a
5′‐CAGTGCGTGTCGTGGAGT‐3′	MiR‐34a‐R	
5′‐UCAGCACCCACCCAGGAAUC‐3′	HOTAIR‐F	Real‐time PCR for HOTAIR
5′‐AGAGUUGCUCUGUGCUGCCA‐3′	HOTAIR‐R	
5′‐TCTGGGGAATAACGCTGGTG ‐3′	PVT‐1‐F	Real‐time PCR for PVT‐1
5′‐CAGCCACAGCCTCCCTTAAA ‐3′	PVT‐1‐R	
5′‐AGCTCCTCGGACAGCTGTAA ‐3′	XIST‐F	Real‐time PCR for XIST
5′‐CTCCAGATAGCTGGCAACC ‐3′	XIST‐R	
5′‐TCAGCTCTGGGATGATGTGGT‐3′	H19‐F	Real‐time PCR for H19
5′‐CTCAGGAATCGGCTCTGGAAG‐3′	H19‐R	
5′‐CGACCACTTTGTCAAGCTCA‐3′	GAPDH‐F	Real‐time PCR for GAPDH
5′‐CCCTGTTGCTGTAGCCAAAT‐3′	GAPDH‐R	

Abbreviations: GAPDH, glyceraldehyde 3‐phosphate dehydrogenase; HOTAIR, homeobox transcript antisense intergenic RNA; PCR, polymerase chain reaction: PVT‐1, plasmacytoma variant translocation 1; XIST, X inactive‐specific transcript.

Then, RT‐PCR was performed on a 20 μL reaction mixture with 10 µL of 2X SYBR Premix Ex Taq, 0.5 μL (10 pmol/μL) of each primer (HOTAIR, PVT‐1, XIST, H19, and GAPDH), 8 μL of nuclease‐free distilled water, and 1 μL of cDNA for analysis of the mentioned lncRNA expression levels. Therefore, RT‐PCR was performed at 95°C for 15 min, which was followed by 40 cycles at 95°C for 15 s, 60°C for 30 s, and finally 72°C for 20 s, using the Rotor‐Gene Q instrument. Further, GAPDH as an internal control was used to normalize HOTAIR, PVT‐1, H19, and XIST expression levels.^42^ The $2^{‐∆∆C_{t}}$ formula was utilized for the calculations of the fold change of selected lncRNAs. Consequently, all the samples were tested in triplicate.

### 2.5 Statistical analyses

SPSS 20.0 and Prism 9.0 software were employed for data analyses.^43^, ^44^ At least three independent measurements of ncRNAs were performed. The assessment of the normality of quantitative variables and homogeneity of error variances were evaluated by the Shapiro–Wilk test and Brown–Forsythe test, respectively.^45^, ^46^ Furthermore, the mean fold change difference of ncRNAs between the study groups was analyzed using two‐tailed Mann–Whitney or Kruskal–Wallis tests.^47^ Moreover, Fisher's exact and Mann–Whitney *U* tests were utilized to determine the statistical difference between the two groups of COVID‐19 subjects. The correlation between the levels of ncRNAs with laboratory data and the clinical features of COVID‐19 subjects was determined via Spearman's correlation coefficient.^48^, ^49^

## 3 RESULTS

### 3.1 Profiles of research units

The current study includes a total of 120 participants, 80 of whom are COVID‐19 patients admitted to Hazrat Rasoul Akram Hospital in Tehran, Iran, and the other 40 are healthy individuals (group 1). According to the findings, the mean age of the participants was calculated to be 36.5 ± 11.9 (ranging between 23 and 68 years) for healthy people, 35.9 ± 10.2 (ranging between 22 and 63 years) for mild COVID‐19 subjects, and 62.5 ± 14.0 (ranging between 18 and 90 years) for severe COVID‐19 subjects (Table 2). Furthermore, in the mild COVID‐19 group, the severe COVID‐19 group, and healthy controls, 20/40 (50%), 26/40 (65%), and 20/40 (50%) were males, respectively (Table 2). Tables 3 and 4 present a summary of the epidemiological and laboratory features of the participants under study.

**Table 2:** Demographic characteristics of the studied participants.

Parameters	Male	Female	Total	*p* Value
Healthy controls individuals (group 1)				
No (%)	20/40 (50%)	20/40 (50%)	40/40 (100%)	‐
Mean age ± SD (range)	38.2 ± 13.5 (23–68)	34.7 ± 10.0 (24–60)	36.5 ± 11.9 (23–68)	0.448 Mann–Whitney *U* test
Nonhospitalized patients with COVID‐19 (group 2)				
No (%)	20/40 (50%)	20/40 (50%)	40/40 (100%)	‐
Mean age ± SD (range)	38.3 ± 8.3 (28–53)	33.6 ± 11.5 (22–63)	35.9 ± 10.2 (23–68)	0.026^a^ Mann–Whitney *U* test
Hospitalized patients with COVID‐19 (group 3)				
No (%)	26/40 (65%)	14/40 (35%)	40/40 (100%)	‐
Mean age ± SD (range)	57.8 ± 13.1 (18–72)	71.6 ± 11.0 (56–90)	62.5 ± 14.0 (18–90)	0.001^a^ Mann–Whitney *U* test

^a^ Statistically significant.

**Table 3:** Clinical characteristics of COVID‐19 participants according to severity group.

Parameters	Group 2^a^	Group 3^b^	*p* Value
Fever	30/40 (75%)	38/40 (95%)	0.02
Chills	17/40 (42.5%)	20/40 (50%)	0.6
Headache	23/40 (57.5%)	33/40 (82.5%)	0.02
Weakness	8/40 (20%)	22/40 (55%)	0.002
Confusion	7/40 (17.5%)	13/40 (32.5%)	0.1
Chest pain	11/40 (27.5%)	25/40 (62.5%)	0.002
Shortness of breath	9/40 (22.5%)	16/40 (40%)	0.1
Dry cough	21/40 (52.5%)	28/40 (70%)	0.1
Sputum cough	3/40 (7.5%)	11/40 (27.5%)	0.01
Skeletal pain	29/40 (72.5%)	36/40 (90%)	0.08
Decreased smell	7/40 (17.5%)	4/40 (10%)	0.5
Decreased taste	6/40 (15%)	4/40 (10%)	0.7
Gastrointestinal symptom	21/40 (52.5%)	23/40 (57.5%)	0.4
Runny nose	13/40 (32.5%)	20/40 (50%)	0.1
Stuffy nose	12/40 (30%)	22/40 (55%)	0.04
Bleeding stomach	2/40 (5%)	6/40 (15%)	0.2

*Note*: All clinical data analyzed by Fisher's exact test between group 2 and group 3.

^a^ Group 2: COVID‐19 outpatients.

^b^ Group 3: COVID‐19 hospitalized patients.

**Table 4:** Laboratory data of COVID‐19 participants according to severity group.

Parameters	Group 2^a^	Group 3^b^	*p* Value^c^
FBS (mg/dL)	84.0 ± 10.3 (69–110)	203 ± 127.4 (77–512)	<0.001
Urea (mg/dL)	21.1 ± 5.8 (14–35)	24.7 ± 11.2 (12–58)	0.4
Cr (mg/dL)	1.1 ± 0.4 (0.5–2.4)	1.5 ± 0.9 (0.8–4.8)	0.001
LDH (U/L)	236.2 ± 94.1 (109–439)	581.3 ± 268.1 (189–1243)	<0.001
CPK (U/L)	59.5 ± 34.2 (23–143)	319.3 ± 685.0 (26–3200)	<0.001
ALP (U/L)	94.1 ± 37.3 (41–140)	306.4 ± 217.0 (96–1037)	<0.001
AST (U/L)	17.3 ± 9.1 (9–33)	62.3 ± 43.4 (10–154)	<0.001
ALT (U/L)	19.5 ± 9.9 (10–39)	61.0 ± 35.0 (11–126)	<0.001
WBC (10^3^/μL)	7.8 ± 1.5 (4.1–10.3)	9.7 ± 6.5 (2.6–32.4)	0.9
RBC (10^6^/μL)	4.4 ± 4.5 (3.4–5.4)	4.4 ± 1.1 (2.4–7.0)	0.6
Hb (g/dL)	13.8 ± 1.2 (11.6–15.4)	13.1 ± 3.3 (7–20)	0.1
Hct (%)	42.0 ± 4.2 (34–49)	39.4 ± 8.5 (23–59)	0.059
INR	1.1 ± 0.1 (0.8–1.3)	1.4 ± 0.9 (1–5.3)	0.004
PTT (S)	31.0 ± 4.2 (25–39)	38.0 ± 18.0 (25–103)	0.03
Platelet count (10^3^/μL)	238 ± 108 (105–437)	194 ± 130 (11–485)	0.02
Ca (mg/dL)	9.9 ± 0.7 (8.9–11.2)	8.7 ± 1.6 (2.7–10.8)	<0.001
Ph (mg/dL)	3.9 ± 0.5 (2.8–4.9)	3.4 ± 0.9 (1.6–4.3)	0.06
Na (mEq/L)	140 ± 3.1 (136–148)	137 ± 5.5 (124‐147)	0.001
K (mEq/L)	4.1 ± 0.5 (3.4–5.3)	4.4 ± 0.6 (3.5–5.7)	0.01
CRP (mg/dL)	8.4 ± 4.0 (2–19)	28.0 ± 13.6 (12–53)	<0.001
Vitamin D (ng/mL)	21.3 ± 10.3 (9–44)	24.0 ± 14.0 (4–45)	0.7

Abbreviations: ALT, alanine transaminase; AST, aspartate aminotransferase; Ca, calcium; CPK, creatine phosphokinase; Cr, creatinine; CRP, C‐reactive protein; FBS, fasting blood sugar; Hb, hemoglobin; Hct, hematocrit; INR, international normalized ratio; K, potassium; LDH, lactate dehydrogenase test; Na, sodium; Ph, potential hydrogen; PTT, partial thromboplastin time; RBC, red blood cell; WBC, white blood cell.

^a^ Group 2: COVID‐19 outpatients.

^b^ Group 3: COVID‐19 hospitalized patients.

^c^ The Mann–Whitney *U* test was used for comparing laboratory data between group 2 and group 3.

### 3.2 LncRNA‐HOTAIR and ‐XIST overexpressed in severe COVID‐19 groups

**Figure 1:**
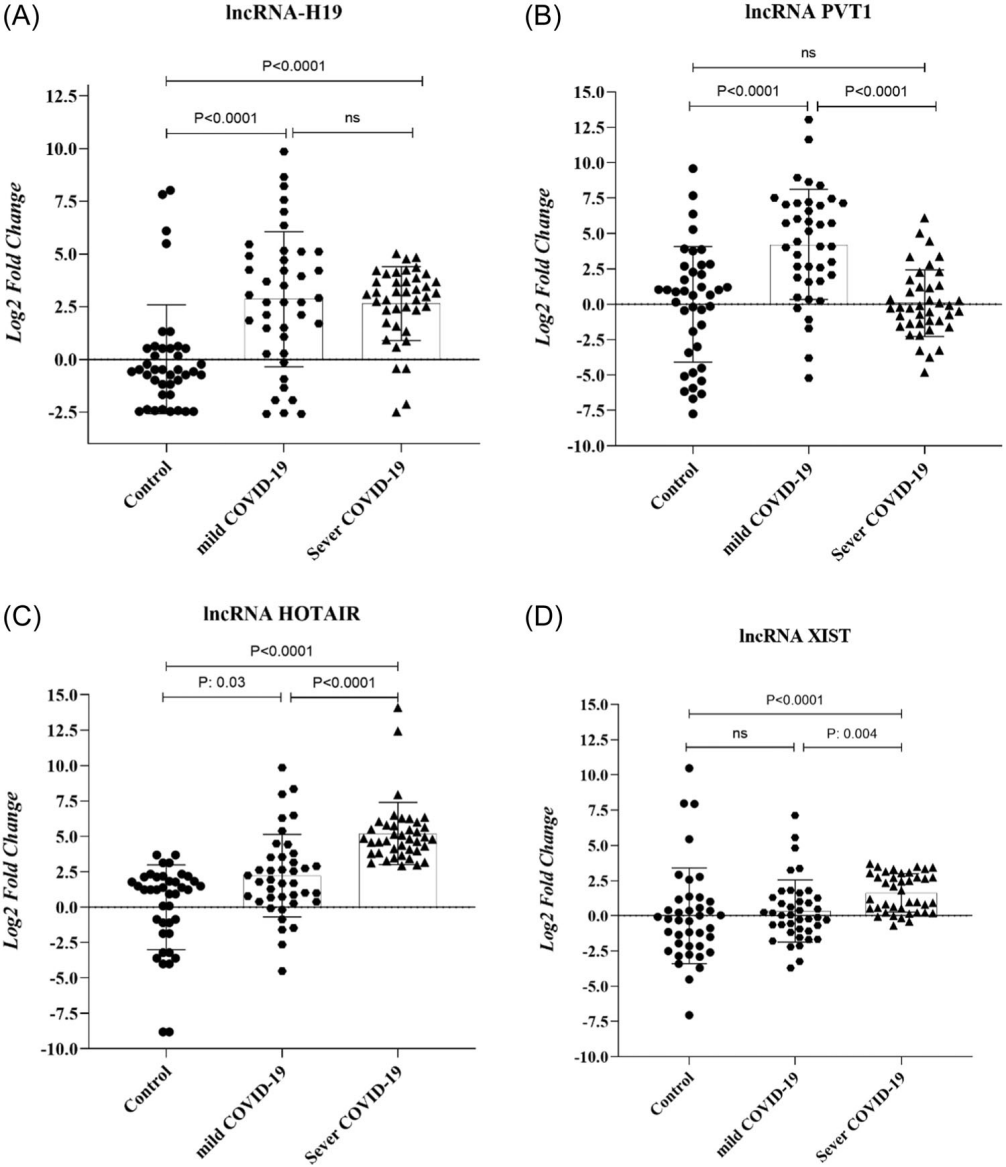
Long noncoding RNAs (lncRNAs), (A) H19, (B) plasmacytoma variant translocation 1 (PVT‐1), (C) homeobox C antisense intergenic RNA (HOTAIR), and (D) X‐inactive‐specific transcript (XIST) levels measured in mild coronavirus disease 19 (COVID‐19) patients (*n* = 40), severe COVID‐19 patients (*n* = 40), and healthy control subjects (*n* = 40). The expression level of lncRNAs demonstrated significant differences among the groups (ns, not significant).

In this step, the expression patterns of the selected lncRNA, including lncRNA‐H19, ‐PVT‐1, ‐HOTAIR, and ‐XIST, were evaluated in the PBMC of the healthy individuals in severe and mild COVID‐19 groups. As shown in Figure 1, significantly elevated levels (shown as mean fold change ± SD) of lncRNA H19 (2.58 ± 3.2 vs. 0 ± 2.5, *p* < 0.0001), PVT‐1 (4.2 ± 3.8 vs. 0 ± 4.08, *p* < 0.0001), and HOTAIR (2.2 ± 2.9 vs. 0 ± 3, *p* = 0.03) were noted in the mild COVID‐19 group versus the healthy controls. Nonetheless, no considerable difference was observed in the lncRNA XIST levels between these samples (Figure 1D). According to the results, lncRNA H19, HOTAIR, and XIST levels in the severe COVID‐19 subjects were remarkably higher than control samples. Detailed information is presented in Table 5.

**Table 5:** The details of descriptive statistics for the log2 fold‐change of lncRNAs‐HOTARI, H19, XIST, PVT‐1, and miR‐34a.

	Groups	HOTARI	H19	XIST	PVT‐1	miR‐34a
Mean	Control	0.002	0.001	0.004	0.001	–0.005
	Mild COVID‐19	2.223	2.858	0.35	4.226	–1.517
	Sever COVID‐19	5.205	2.648	1.639	0.08	1.094
Std. deviation	Control	3	2.582	3.402	4.087	1.719
	Mild COVID‐19	2.924	3.203	2.215	3.885	2.321
	Sever COVID‐19	2.197	1.756	1.365	2.358	1.770
Std. error of mean	Control	0.474	0.408	0.537	0.646	0.271
	Mild COVID‐19	0.462	0.506	0.35	0.614	0.367
	Sever COVID‐19	0.347	0.277	0.215	0.372	0.279
Median	Control	1.24	–0.53	–0.275	0.765	–0.05
	Mild COVID‐19	1.872	2.81	0.045	4.255	–1.84
	Sever COVID‐19	4.815	3.115	1.5	–0.25	0.995
Range	Control	12.5	10.51	17.53	17.34	7.88
	Mild COVID‐19	14.37	12.45	10.83	18.26	11.66
	Sever COVID‐19	11.17	7.520	4.4	10.91	8.490
Minimum	Control	–8.81	–2.48	–7.05	–7.75	–3.1
	Mild COVID‐19	–4.517	–2.59	–3.7	–5.21	–8.04
	Sever COVID‐19	2.93	–2.5	–0.7	–4.81	–3
Maximum	Control	3.69	8.02	10.48	9.59	4.78
	Mild COVID‐19	9.85	9.86	7.13	13.05	3.62
	Sever COVID‐19	14.1	5.02	3.7	6.1	5.49

Abbreviations: COVID‐19, coronavirus disease 2019; HOTARI, homeobox C antisense intergenic RNA; lncRNA, long noncoding RNA; PVT‐1, plasmacytoma variant translocation 1; XIST, X inactive‐specific transcript.

**Figure 2:**
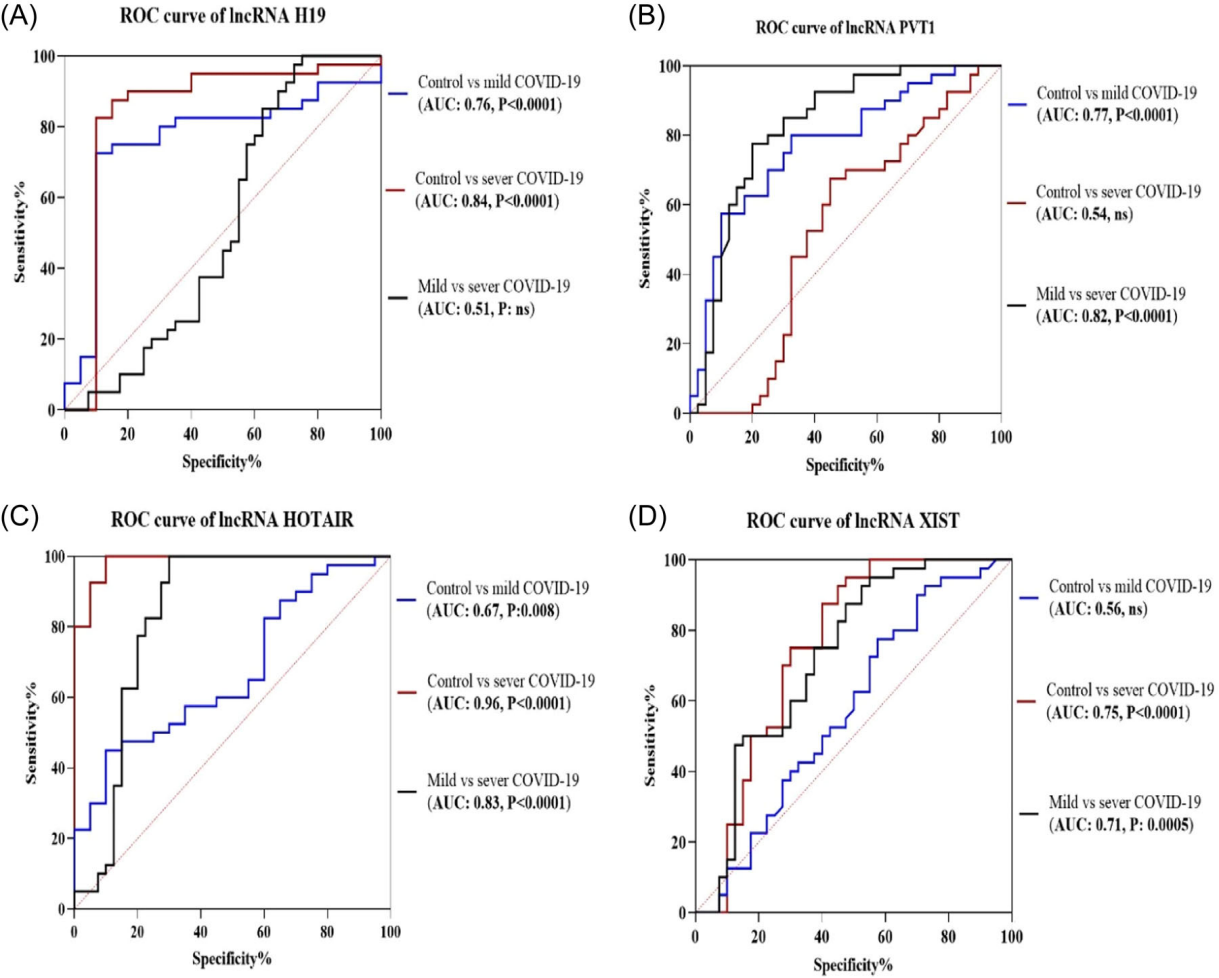
Receiver operating characteristic (ROC) curve of long noncoding RNAs (lncRNAs), (A) H19, (B) plasmacytoma variant translocation 1 (PVT‐1), (C) homeobox C antisense intergenic RNA (HOTAIR), and (D) X‐inactive‐specific transcript (XIST), for discrimination between mild coronavirus disease 2019 (COVID‐19) patients, severe COVID‐19 patients, and healthy control subjects (ns, not significant)‌. The ROC curve analysis demonstrated that the mentioned lncRNAs may act as biomarkers in discriminating COVID‐19 patients from healthy people.

Based on the findings, it was found that the group with severe COVID‐19 had a notably higher level of lncRNA PVT‐1 expression compared to the group with mild COVID‐19 (Figure 1B, *p* < 0.0001). Additionally, in the group with severe COVID‐19, the mean fold change of lncRNAs HOTAIR and XIST was significantly higher than in mild COVID‐19 patients (5.2 ± 2.1 vs. 2.2 ± 2.9, *p* < 0.0001 and 1.6 ± 1.3 vs. 0.3 ± 2.2, *p* = 0.003, respectively). In addition, the diagnostic potential of the PBMC level of lncRNAs in discriminating between individuals with severe and mild COVID‐19, as well as discriminating between healthy and COVID‐19 patients, was assessed using receiver‐operating characteristic (ROC) analysis (Figure 2). Analyses revealed that PVT‐1, HOTAIR, and XIST may result in the discrimination of severe COVID‐19 from mild COVID‐19 (the area under the curve [AUC] of 0.82, sensitivity of 82.5%, specificity of 62.5%, and *p* < 0.0001; AUC of 0.83, specificity of 77.5%, sensitivity of 82.5%, and *p* < 0.0001; AUC of 0.7, sensitivity of 75%, specificity of 62.5%, and *p* < 0.001, respectively). Further, ROC results suggested that H19 and PVT‐1 have an AUC of 0.76, a sensitivity of 80%, a specificity of 70%, and a *p* < 0.0001, as well as an AUC of 0.77, a sensitivity of 77.5%, a specificity of 67.5%, and a *p* < 0.0001, respectively. In addition, the predictive values to discriminate mild COVID‐19 patients from controls were for HOTAIR (AUC of 0.96) and for H19 (AUC of 0.84).

The correlation was assessed between lncRNAs with clinical characteristics and laboratory data of patients with COVID‐19 using the Spearman correlation tests (Table 6). Correlation analysis results showed that the fold changes in the lncRNA HOTAIR in patients with COVID‐19 were in a significant positive correlation with CRP (*r* = 0.55, *p* < 0.0001), alkaline phosphatase (*r* = 0.32, *p* = 0.009), aspartate transaminase (*r* = 0.36, *p* = 0.006), alanine transaminase (*r* = 0.39, *p* = 0.002), lactate dehydrogenase (*r* = 0.43, *p* < 0.001), fasting blood glucose (*r* = 0.46, *p*  <  0.001), diabetes (*r* = 0.35, *p* = 0.007), gastrointestinal symptom (*r* = 0.35, *p* = 0.007), runny nose (*r* = 0.36, *p* = 0.006), stuffy nose (*r* = 0.39, *p* = 0.002), headache (*r* = 0.41, *p* < 0.001), fever (*r*  = 0.43, *p* < 0.001), and weakness (*r* = 0.31, *p* = 0.01). Moreover, a significantly positive association was observed between miR‐34a and diabetes (*r* = 0.37, *p* = 0.005) and FBS (*r* = 0.44, *p* < 0.001). Detailed information is provided in Table 6.

**Table 6:** Correlation between noncoding RNAs with laboratory data and clinical characteristics of COVID‐19 patients.

	H19	PVT‐1	HOTAIR	XIST	miR‐34a
Diabetes	0.10	–0.08	0.35**	0.18	0.18
Fever	0.35**	0.18	0.43***	0.13	0.16
Weakness	0.17	0.01	0.31**	0.11	0.16
Confusion	0.08	0.08	0.16	0.06	0.11
Headache	0.27	–0.04	0.41***	0.20	0.19
Chills	0.23	0.05	0.28	0.17	0.00
Skeletal pain	0.24	0.11	0.36	0.11	0.14
Dry cough	0.18	0.06	0.28	0.05	0.26
Sputum cough	0.03	–0.05	0.24	0.08	0.16
Chest pain	0.09	–0.11	0.24	0.05	0.26
Shortness of breath	0.07	–0.03	0.21	0.06	0.14
Runny nose	0.23	0.04	0.36**	0.19	0.09
Stuffy nose	0.28	0.12	0.39**	0.15	0.00
Decreased smell	–0.03	0.04	–0.08	–0.05	–0.09
Decreased taste	0.15	0.13	0.16	0.09	–0.06
Gastrointestinal symptom	0.26	0.15	0.35**	0.12	0.21
Bleeding stomach	–0.04	–0.06	0.14	0.02	0.11
WBC (10^3^/μL)	0.14	0.02	0.16	0.11	0.18
RBC (10^6^/μL)	0.12	–0.06	0.16	0.03	0.00
Hb (g/dL)	0.15	0.08	–0.02	0.00	–0.07
Hct (%)	0.15	0.12	–0.04	0.02	–0.10
Platelet count (10^3^/μL)	–0.24	0.00	–0.14	–0.05	–0.13
INR	0.04	0.11	0.10	0.08	0.05
PTT (S)	0.10	–0.04	0.11	0.02	0.12
FBS (mg/dL)	0.05	–0.05	0.46***	0.00	0.12
Urea (mg/dL)	0.11	–0.11	0.04	0.06	0.13
Cr (mg/dL)	0.07	–0.06	0.13	0.20	0.28
AST (U/L)	0.05	–0.11	0.36**	0.23	0.33**
ALT (U/L)	0.16	–0.11	0.39**	0.23	0.25
LDH (U/L)	0.13	0.03	0.43***	0.09	0.13
CPK (U/L)	0.09	–0.10	0.16	0.15	0.28
ALP (U/L)	–0.15	–0.12	0.32**	–0.16	–0.01
Na (mEq/L)	–0.16	–0.45***	–0.18	–0.04	0.35**
K (mEq/L)	–0.12	0.16	0.14	–0.10	–0.32
Ca (mg/dL)	–0.13	0.06	–0.22	–0.14	–0.14
Ph (mg/dL)	0.14	–0.14	–0.28	0.23	0.30
CRP (mg/dL)	–0.08	–0.12	0.55****	0.08	0.09
Vitamin D (ng/mL)	0.10	–0.08	0.02	0.18	0.18

Abbreviations: ALP, alkaline phosphatase; ALT, alanine transaminase; AST, aspartate aminotransferase; Ca, calcium; COVID‐19, coronavirus disease 2019; CPK, creatine phosphokinase; Cr, creatinine; CRP, C‐reactive protein; FBS, fasting blood sugar; Hb, hemoglobin; Hct, hematocrit; HOTARI, homeobox C antisense intergenic RNA; INR, international normalized ratio; K, potassium; LDH, lactate dehydrogenase test; Na, sodium; Ph, potential hydrogen; PTT, partial thromboplastin time; PVT‐1, plasmacytoma variant translocation 1; RBC, red blood cell; WBC, white blood cell; XIST, X inactive‐specific transcript

* *p* < 0.05

** *p* < 0.01

*** *p* < 0.001

**** *p* < 0.0001.

### 3.3 Negative correlation between PVT‐1 and miR‐34a

**Figure 3:**
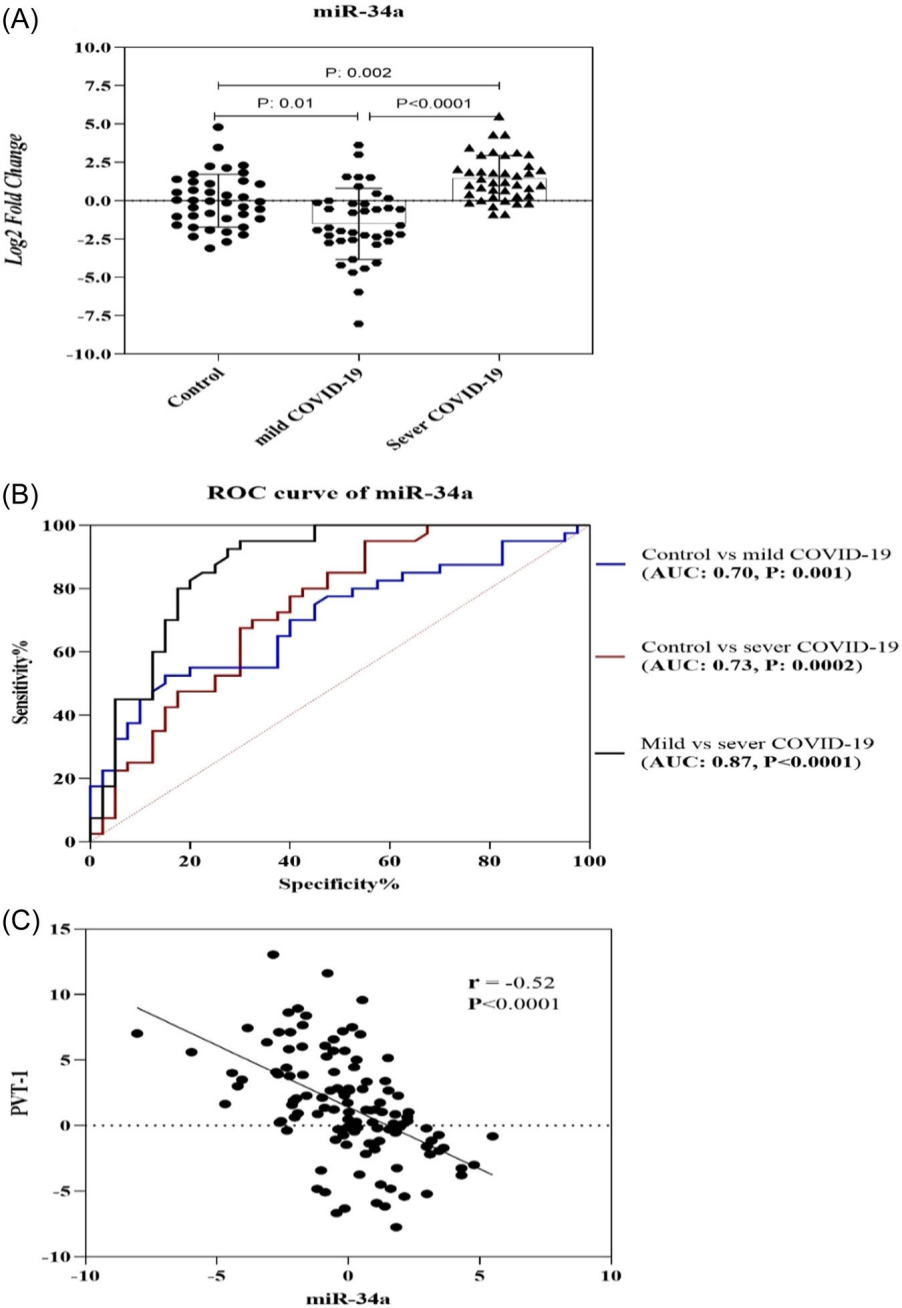
Comparison of the expression levels of miR‐34 Between study groups (A), receiver operating characteristic (ROC) curve analysis of miR‐34a (B), and correlation of miR‐34a and long noncoding RNA (lncRNA)‐plasmacytoma variant translocation 1 (PVT‐1). The expression level of miR‐34a was significantly upregulated in severe coronavirus disease 19 (COVID‐19) patients compared to the other study group (A). In addition, there was a significant negative correlation between the level of miR‐34a and lncRNA‐PVT‐1 (C). AUC, area under the curve.

Considering Figure 3A, miR‐34a remarkably decreased in the mild COVID‐19 group compared with healthy subjects (mean fold change ± SD: −1.51 ± 2.32 vs. −0.002 ± 1.71, *p* = 0.01). On the other hand, in the severe COVID‐19 group, the mean fold change of miR‐34a was significantly higher than that of control (1.45 ± 1.48 vs. −0.002 ± 1.71; *p* = 0.002) and patients with mild COVID‐19 (1.45 ± 1.48 vs. −1.51 ± 2.32; *p* < 0.0001).

According to the results of the ROC curve, miR‐34a may be the most important biomarker for distinguishing severe COVID‐19 from mild COVID‐19 in our study (AUC: 0.87, *p* < 0.0001, Figure 3B). Additionally, miR‐34a expression levels contribute to discrimination between healthy individuals and those in the mild COVID‐19 phase (AUC: 0.70, *p* = 0.001). Further, this miRNA can be useful to discriminate between the healthy control group and the severe COVID‐19 group (AUC: 0.73, *p* = 0.0002). Furthermore, according to correlation results, a significant positive correlation was found between the miR‐34 level and AST and Na levels (for both *r* = 0.35, *p* = 0.007).

Some lncRNAs are responsible for the regulation of mRNA expression via binding to miRNAs and act as competitive endogenous RNAs.^50^, ^51^ In this research, a reverse correlation was observed between PVT‐1 and miR‐34a (*r* = −0.52, *p* < 0.0001, Figure 3C).

## 4 DISCUSSION

The present research analyzed the expression patterns of selected lncRNAs (HOTAIR, H19, PVT‐1, and XIST) and miR‐34a among healthy control, mild COVID‐19, and severe COVID‐19 groups. Then, the levels of the selected ncRNAs were specified, showing a significant difference between the study groups. Furthermore, lncRNA HOTAIR levels were correlated with inflammatory factors such as CRP.

Increasing research represents that lncRNAs may act as regulators of inflammatory pathways and can play major roles in inflammation‐related disorders and hyperinflammation.^20^, ^52^, ^53^ The cross‐talking among ncRNAs and their target mRNAs involved in inflammation leads to regulating the responses of the host immune system.^20^, ^27^, ^54^ It has been reported that ncRNAs can affect different stages of the viral cycle and the host's immune responses. In return, viruses regulate ncRNA expression to facilitate their own replication cycle.^55^, ^56^, ^57^ Recently, it has been observed that some ncRNAs can target the genome or some genes of SARS‐CoV‐2.^58^, ^59^, ^60^ For example, Natarelli et al.^61^ suggested that lncRNAs H19 and HOTAIR probably could potentially bind to the SARS‐CoV‐2 S coding region.^61^ Moreover, another study has reported a strong affinity interaction between HOTAIR and the SARS‐CoV‐2 genome.^62^ Therefore, these lncRNAs can serve as novel agents for inhibiting or regulating SARS‐CoV‐2 gene expression. Hence, the results demonstrated that HOTAIR and H19 levels in severe and mild COVID‐19 groups are higher than in the control. Interestingly, in the PBMC of severe COVID‐19 cases, the levels of HOTAIR are greater than in mild COVID‐19 cases. Considering that the HOTAIR level was remarkably overexpressed in the group with severe COVID‐19 in comparison with the group with mild COVID‐19 and the healthy group, the interaction between HOTAIR and the SARS‐CoV‐2 genome probably has no inhibitory effect on viral replication. Additionally, ROC analysis demonstrated that HOTAIR can be utilized as one of the new biomarkers for discriminating severe COVID‐19 from mild COVID‐19 (AUC: 0.83, *p* < 0.0001) and healthy subjects (AUC: 96, *p* < 0.0001). However, there is a need for experimental research to clarify the function of HOTAIR during viral replication and the biomarker role of this lncRNA.

On the other hand, hyper‐inflammation and cytokine storms have been proposed to be the most prominent immunopathology in severe COVID‐19.^63^ Interleukin (IL)‐6 plays a major role in viral cytokine storms during COVID‐19.^64^ Interestingly, lncRNA HOTAIR can induce nuclear factor kappa B (NF‐κB) activation and lead to promoting IL‐6 expression levels, and in return, knock‐down of HOTAIR leads to reduced inflammation levels.^27^ In addition, H19 may be involved in inflammation development by regulating inflammatory pathways.^65^, ^66^, ^67^ Along these lines, a remarkable positive correlation was found among the levels of HOTAIR with inflammation‐related factors, such as CRP, lactate dehydrogenase test (LDH), and fever, in COVID‐19 subjects, whereas no significant correlation was detected between H19 and inflammation‐related factors (Table 6). Hence, to overcome this limitation, investigations with larger sample sizes must confirm the diagnostic values of such lncRNAs.

Studies in the field have identified lncRNA‐XIST as the initiator of inactivating the X chromosome, and the deregulation of XIST expression levels has been found in several sex‐biased diseases.^68^ Reportedly, XIST can modulate inflammation by regulating the signaling pathway NF‐κB.^69^ Recently, Cheng et al.^70^ found that the XIST levels in the PBMC of the group with severe COVID‐19 (*n* = 17) were remarkably downregulated in comparison with the group with nonsevere COVID‐19 (*n* = 12). In another research, Askari et al.^71^ reported that XIST levels in both intensive care unit (ICU) and non‐ICU men with COVID‐19 were higher than those in women with COVID‐19.^71^ Consistent with their results, our study findings demonstrated that in the severe COVID‐19 group, the level of XIST was considerably greater than in the mild COVID‐19 group and healthy groups. In addition, the expression of XIST was overexpressed in the males who suffered from severe COVID‐19 in comparison to the women suffering from mild COVID‐19 (*p* = 0.02). Although no differences were observed in the COVID‐19 incidence between men and women, studies have shown that men are more susceptible to severe SARS‐CoV‐2 infection, and higher severity and fatality rates in men are higher than in women.^72^, ^73^ Hence, one of the factors that can be considered more in males with severe COVID‐19 is lncRNA XIST, which requires more studies.

One of the conserved miRNAs in mammals is miR‐34a, which is deregulated in various human diseases, such as cancer.^74^ An important role of miR‐34a in inflammatory regulation has been reported in several studies.^34^, ^35^, ^75^, ^76^ For example, miR‐34a can attenuate inflammation by suppressing the NF‐κB pathway, which, as a result, causes decreased lung injury.^77^, ^78^, ^79^ Further, the downregulation of the miR‐34 expression level in the airway or lung tissue samples of patients with COVID‐19 was reported in some studies.^80^, ^81^ Unlike these studies, our results demonstrated that the PBMC level of miR‐34a was remarkably upregulated in the group with severe COVID‐19. However, a highly downregulated level of miR‐34a was observed in the group with mild COVID‐19. Such different results may be due to the progression of SARS‐CoV‐2 infection, different clinical phases of COVID‐19, and/or different host immune system responses in different tissues.

According to the bioinformatics analysis result, PVT‐1, HOTAIR, XIST, and H19 can be the target miR‐34a. However, according to Spearman correlation results, only a significant negative association was found between miR‐34a and PVT‐1 in the study subjects (Figure 3). Both PVT‐1 and miR‐34a are involved in inflammatory pathways^34^, ^82^, ^83^, ^84^; thus, it is likely that PVT‐1 is involved in the regulation of inflammatory responses by targeting miR‐34a during the infection of SARS‐CoV‐2. According to ROC curve results, lncRNA HOTAIR and miR‐34a can be utilized as potential prognostic biomarkers in inflammatory status in COVID‐19 subjects and diabetic COVID‐19 subjects.

In conclusion, a remarkable difference in the expression level of the selected lncRNAs, including HOTAIR, H19, XIST, PVT‐1, and miR‐34a, was observed between COVID‐19 groups. Moreover, to the best of our knowledge, this is the first report in this field, and the findings revealed that HOTAIR and H19 expression were considerably increased in the groups with severe COVID‐19 versus the healthy group and the group with mild COVID‐19. Considering that there is a positive correlation between HOTAIR levels and inflammation‐related factors such as CRP, LDH, and fever, this lncRNA may act as an inflammatory response‐related prognostic biomarker in cases of COVID‐19. The lncRNA XIST level, which is required for the inactivation of the X chromosome in the cells of a female mammal,^68^ is lower in women with mild COVID‐19 in comparison to men with severe COVID‐19. LncRNA XIST is probably one of the factors that could be involved in the development of severe COVID‐19 in men. A negative correlation between lncRNA PVT‐1 and miR‐34a and the role of both of these factors in the regulation of inflammatory pathways^34^, ^82^, ^83^, ^84^ evoke the idea that PVT‐1 can control the progression of inflammation in COVID‐19 patients via targeting miR‐34a. Taken together, the aberrant level of expression of the selected factors in the cases with COVID‐19 and ROC curve analysis results demonstrated that these factors can probably contribute to the pathogenicity of SARS‐CoV‐2, which can be considered diagnostic biomarkers for patients with mild or severe COVID‐19. Nonetheless, our research is followed by limitations such as the limited number of samples and the investigation of these ncRNAs only in PBMC samples, which may have affected our findings. Hence, more studies are required to confirm these results.

## AUTHOR CONTRIBUTIONS

**Khadijeh Khanaliha**: Data curation; investigation; methodology. **Javid Sadri Nahand**: Formal analysis; investigation; methodology; software. **AliReza Khatami**: Methodology; writing—original draft. **Hamed Mirzaei**: Investigation; methodology. **Sara Chavoshpour**: Software; writing—original draft. **Mohammad Taghizadieh**: Data curation; writing—review and editing. **Mohammad Karimzadeh**: Formal analysis; software. **Tahereh Donyavi**: Writing—original draft. **Farah Bokharaei‐Salim**: Supervision.

## CONFLICT OF INTEREST STATEMENT

The authors declare no conflict of interest.

## TRANSPARENCY STATEMENT

The lead author Farah Bokharaei‐Salim affirms that this manuscript is an honest, accurate, and transparent account of the study being reported; that no important aspects of the study have been omitted; and that any discrepancies from the study as planned (and, if relevant, registered) have been explained.

## Data Availability

The authors have nothing to report.
